# Supplementary material for: The critical factor: The role of quality in the performance of supported accommodation services for complex mental illness in England
Source: PLoS One. 2022 Mar 17;17(3):e0265319. doi: 10.1371/journal.pone.0265319 (PMC8929565; doi:10.1371/journal.pone.0265319)
Supplement: Attachment — Submitted filename: Response to the reviewers.docx [file pone.0265319.s001.docx]

Dear editor,

Many thanks for your comments, we highly appreciate your suggestions and questions. According to them, we have carried out the corresponding changes in the paper (highlighted in yellow).

Sincerely,

The authors.

**EDITOR’S COMMENTS**

Comment nº 1.

**The abstract is loosely written. A standard abstract must present, without leaving any doubt, the objective of the paper precisely; source of data (which is not present in your abstract) and analytical approach used; key findings and any policy implication and recommendations.**

Thank you for your recommendation. A new abstract has been included following the proposed structure.

Comment nº 2.

**The arguments are fairly presented but the statement that justifies the study does not come clearly (i.e. Why did you started this research?).**

According to your kind suggestion, the following paragraph has been included in the introduction:

*The standard performance assessment of any kind of comparable services includes input consumption and output production (directly related to the inputs consumed). These inputs/outputs are mainly technical. The incorporation of quality variables (in this study representing by six/seven quality domains) is always complicated because they are perceptions (perceived quality from users, managers, etc.). To our knowledge, this is the first study that includes quality domains, estimated by the managers of the selected MH services, into a performance analysis (6). Independently of the technical performance of the services (the baseline scenario analysed without quality domains) the main research question is to assess the impact on the service performance when quality domains are incorporated into the analysis. Taking into account that MH service managers have a specific amount of inputs and always try to obtain the corresponding best results, a neutral/positive relationship between managerial processes (input and output management) and their quality perceptions on performance is expected.*

Other changes include a relevant modification of the following paragraph:

*Relative Technical Efficiency (RTE) is a decision support technique that can be used for guiding health informed evidence-based policy-making, mainly on improving resources allocation (7,8). RTE assesses the relationship among the amount of inputs consumed and outputs produced by a set of comparable decision making units (9). RTE can be regarded as a synthetic meta-indicator that facilitates monitoring the evolution of a system and the dynamic relationships or connections across different performance indicators (10,11). It has been used for identifying tailor-made improvement strategies for health care ecosystems like provision and resourcing of addiction treatment clinics (12,13), residential MH facilities (14), homes for people with mental disability (15), clinics for children and youth (16) and community based youth services (17). The RTE of primary care and MH ecosystems have been systematically assessed in the Basque Country (Spain) (5,18–20). Data Envelopment Analysis (DEA) has been widely used to assess the RTE of health services (6,18,21). This group of non-parametric techniques are very robust and flexible because they don´t need any preliminary assumption on the variable statistical structure. This means that variables (inputs/outputs) from different origins and types (e.g. number of beds, technical input, and quality of care, manager perception) can be analysed at the same time. Quality variables have been included in DEA for assessing the RTE of systems operating in different socio-economic contexts (e.g. hospital care, schools or the banking industry) (22–25).*

*DEA can be included in a Monte Carlo simulation engine to include uncertainty and randomness in data values (all of them are considered statistical distributions) and design more realistic models (5). Being DEA an operational model, it is completely blind. Variable values must be interpreted according to the existing expert knowledge (usually a theoretical paradigm). A fuzzy inference engine allows to make operational the formalization of the Balanced Care model following the EbCA methodology (5). This engine interprets variable values before RTE was calculated.*

Comment nº 3.

**The introduction does not precisely construct the research problem tackled and does not show how the problem is taken care.**

Thank you for your comment. For that reason, we have included the following paragraph (see also the previous comment):

*The standard performance assessment of any kind of comparable services includes input consumption and output production (directly related to the inputs consumed). These inputs/outputs are mainly technical. The incorporation of quality variables (in this study representing by six/seven quality domains) is always complicated because they are perceptions (perceived quality from users, managers, etc.). To our knowledge, this is the first study that includes quality domains, estimated by the managers of the selected MH services, into a performance analysis (6). Independently of the technical performance of the services (the baseline scenario analysed without quality domains) the main research question is to assess the impact on the service performance when quality domains are incorporated into the analysis. Taking into account that MH service managers have a specific amount of inputs and always try to obtain the corresponding best results, a neutral/positive relationship between managerial processes (input and output management) and their quality perceptions on performance is expected.*

Comment nº 4.

**Why did you used MC simulation, DEA and fuzzy system in the study? These techniques also should be discussed.**

Thanks again, according to this comment we have modify the following paragraph in the introduction (see also comment 2):

*Relative Technical Efficiency (RTE) is a decision support technique that can be used for guiding health informed evidence-based policy-making, mainly on improving resources allocation (7,8). RTE assesses the relationship among the amount of inputs consumed and outputs produced by a set of comparable decision making units (9). RTE can be regarded as a synthetic meta-indicator that facilitates monitoring the evolution of a system and the dynamic relationships or connections across different performance indicators (10,11). It has been used for identifying tailor-made improvement strategies for health care ecosystems like provision and resourcing of addiction treatment clinics (12,13), residential MH facilities (14), homes for people with mental disability (15), clinics for children and youth (16) and community based youth services (17). The RTE of primary care and MH ecosystems have been systematically assessed in the Basque Country (Spain) (5,18–20). Data Envelopment Analysis (DEA) has been widely used to assess the RTE of health services (6,18,21). This group of non-parametric techniques are very robust and flexible because they don´t need any preliminary assumption on the variable statistical structure. This means that variables (inputs/outputs) from different origins and types (e.g. number of beds, technical input, and quality of care, manager perception) can be analysed at the same time. Quality variables have been included in DEA for assessing the RTE of systems operating in different socio-economic contexts (e.g. hospital care, schools or the banking industry) (22–25).*

Additionally, we have modified the following paragraph:

*DEA can be included in a Monte Carlo simulation engine to include uncertainty and randomness in data values (all of them are considered statistical distributions) and design more realistic models (5). Being DEA an operational model, it is completely blind. Variable values must be interpreted according to the existing expert knowledge (usually a theoretical paradigm). A fuzzy inference engine allows to make operational the formalization of the Balanced Care model following the EbCA methodology (5). This engine interprets variable values before RTE was calculated.*

Comment nº 5.

**The research hypotheses’ are not mentioned in the introduction or clear in the literature review.**

Based on your comment, here you can see an additional explanation:

Based on original data from the QUEST project (Killaspy et al., 2017) and a previous systematic review about DEA models in mental health (Garcia-Alonso et al., 2019), this study tries to check, if it exists, the relationship between “… *managerial processes (input and output management) and their* -mental health service managers- *quality perceptions on performance …*”.

Taking into account this comment and according to previous ones, we have included the following paragraph:

*The standard performance assessment of any kind of comparable services includes input consumption and output production (directly related to the inputs consumed). These inputs/outputs are mainly technical. The incorporation of quality variables (in this study representing by six/seven quality domains) is always complicated because they are perceptions (perceived quality from users, managers, etc.). To our knowledge, this is the first study that includes quality domains, estimated by the managers of the selected MH services, into a performance analysis (6). Independently of the technical performance of the services (the baseline scenario analysed without quality domains) the main research question is to assess the impact on the service performance when quality domains are incorporated into the analysis. Taking into account that MH service managers have a specific amount of inputs and always try to obtain the corresponding best results, a neutral/positive relationship between managerial processes (input and output management) and their quality perceptions on performance is expected.*

Comment nº 6.

**Literature review and critical analysis of theories, practices or commentary focusing on existing documents. The study lacks clear description of the literature review:**

Thanks again, we have modified the introduction trying to make it clearer:

1. Explaining the relevance of the situation.
2. Introducing the relevance of integrating quality variables into the technical performance analysis of mental health services and introducing our main hypothesis.
3. Exploring the techniques in an integrated way.
4. Introducing the objective in a clearer way, as follows:

*This study aims to assess the impact of quality indicators (managerial perspective) in the performance (RTE) of selected MH supported accommodation services in the English pathway of care. This objective includes the formalization of specific quality domains into variables (rates), their integration in RTE assessment, and a comparative impact analysis of quality variables on the ecosystem performance to support decision-making and investment by providing relevant information for service managers to inform practice and service planning.*

In all of these sections, relevant literature has been reviewed and explained.

Comment nº 7.

**What I am missing is a description of the review. Did you conduct a systematic literature review? Which years? Key words? What was the literature you found?**

Sorry again, we have modified the objective of the paper because the introduction offered a wrong perspective of our analysis:

*This study aims to assess the impact of quality indicators (managerial perspective) in the performance (RTE) of selected MH supported accommodation services in the English pathway of care. This objective includes the formalization of specific quality domains into variables (rates), their integration in RTE assessment, and a comparative impact analysis of quality variables on the ecosystem performance to support decision-making and investment by providing relevant information for service managers to inform practice and service planning.*

No systematic review was conducted for the present paper.

Comment nº 8.

**Can you better describe how you came to your major variables? You have them from the literature review, but how was literature screened to derive these factors. Methodology and scope of work.**

The original dataset was collected from the QuEST study (raw data). In order to compare the selected MH services original data have been transformed into rates in order to eliminate the “size” effect. For example, it is not the same to have a budget of 100,000 pounds if you have 10 or 100 places or beds. All these rates were discussed several times by experts according to the EbCA model.

The following sentence has been included in the “Scenarios” section:

*All the considered transformations of original data make the selected services comparable by eliminating the potential “size” effect on performance assessment.*

The rates included in this study provide a high reliability of measurement. Additionally, the large number of previous attempts to reach optimal representation of the data allowed us to find differences between residential services: move on and non-move on oriented. According to this, we have included the following sentence in the “Relative Technical Efficiency” section.

*On the other hand, the analytical process of setting the measurement units for the variables highlighted the existence of two different groups in the residential care services dataset: move on and non-move on oriented.*

Comment nº 9.

**The author has poorly discussed the results of the paper. One would expect to find the previous empirical work enriching the discussions of the results, but unfortunately, that has not been done.**

According to your kind suggestion we have added additional comments in the “Results” section:

*Globally speaking, the inclusion of quality domains highlights a neutral-positive or positive impact on the performance of supported accommodation services in England. This is especially relevant in some services that showed lower RTE on average: 13, 18 and 21 (33.3%, Table 3); 14, 90, 96 and 124 (30,8%, Table 4); 18, 22, 27, 58, 70, 97, 110 and 115 (23.5%, Table 5); and, finally, 1, 7, 36, 56, 87, 109, 121 and 122 (26,7%, Table 6). In these services the manager´s perception on quality provided by their services surpass technical results (baseline scenario), probably because there are non-evaluated variables or circumstances in the care provision.*

*On the other hand, quality variables decrease service performance only in supported accommodation services 15, 31, 32, 86 and 98 (14.7%, Table 5). This behaviour can be considered strange because here the manager´s perception on quality provided by their services underestimates their own technical results. Again, there will be additional non-assessed variables or circumstances that can affect the manager´s way of processing information to conclude in detecting frameworks of improving quality of care.*

*Services where the inclusion of quality variables results in a neutral behaviour in their performance are the majority, respectively: 66.7%, 69,2%, 61,8% and 73,3%. In these cases, the manager´s perception on quality provided by their corresponding services matches with their technical results. They obtain a quality according to their resources and outcomes.*

Additionally, the “Discussion” section has been enriched including the following sentences:

*A recent national survey of MH supported accommodation services across England found that the quality of care was higher in MH supported housing than in residential care or floating outreach services (4). Taking into account that quality variables have not included in any RTE assessment of MH services and ecosystems (6,13,15,18,43), the question to answer here is if the perception (given by the corresponding managers) about the quality provided by the MH service is aligned or not with its technical performance. If the impact on RTE scores is negative, then the perception of the quality provided is not aligned with the technical performance (baseline RTE). In this situation, technical results do not achieve an, at least, equivalent quality, according to the manager´s opinion. If the impact can be considered neutral, then technical results and quality perception are balanced. Finally, if it is positive, managers know that the quality proved by their services is better than the corresponding neutral, probably they manage other variables that could not gathered in the QuEST study.*

*… Quality variables increased (sometimes not significantly) RTE on average in a relevant number of the selected services* [residential houses]*, but the majority showed a neutral-positive profile. Quality indicators included non-technical but real service characteristics estimated by the respective managers that, in the end, improved service performance scores.*

*… In this type of care* [supported houses] *a relatively relevant number of services decrease performance scores when quality variables are included. This situation probably indicates that managers take into consideration other variables or service characteristics that end into a poorer quality results than they were expected considering the corresponding resource level and outcome production.*

*… As happens in residential houses, a relevant number of floating outreach services increased their performance scores significantly. Again, this increase is not aligned with the service management (resources and outcomes) showed in the baseline scenario.*

Also in the “Conclusion” section

*Services that have a significant increase or decrease of their performance scores when quality variables are included into the analysis, should be studied. The observed differences to the baseline scenario (only technical) must be a consequence of something related to specific structural o managerial characteristics. These characteristics are a crucial source of information to design new interventions, policies or strategies in order to improve MH care.*
