# Supplementary material for: The critical factor: The role of quality in the performance of supported accommodation services for complex mental illness in England
Source: PLoS One. 2022 Mar 17;17(3):e0265319. doi: 10.1371/journal.pone.0265319 (PMC8929565; doi:10.1371/journal.pone.0265319)
Supplement: Attachment — Submitted filename: Response to Reviewers.docx [file pone.0265319.s002.docx]

Response to Reviewers

Dear Editor and Reviewers, many thanks for your feedback, we have carried out all the changes proposed, which are highlighted in yellow in the “Revised Manuscript with Track Changes”. In this document we have developed a rebuttal letter that responds to each point raised.

**Data availability**

We have uploaded the dataset to Dryad Digital Repository, and it will be available for the public once the article has been published. In addition, Dryad has provided a temporal link for reviewers so they can access to the dataset. This is the link: <https://datadryad.org/stash/share/1eXNj4HhRp0ztCLfgzkwO53k7Rh8q0MvVeZh_A14-xs>

Nevertheless, in the text, we should maintain the original DOI for public access because the reviewers link is temporal and only for revision process. In the text:

*The dataset is available at the Dryad digital repository (*[*https://doi.org/10.5061/dryad.j0zpc86dz*](https://doi.org/10.5061/dryad.j0zpc86dz)*).* (Methods, “Setting” section).

**Reviewer #1: I didn't review the paper in the previous round, but I think the reviewer has pointed the major issues that were existing in the manuscript. In my opinion, the authors have solved issues properly. Based on that I think the paper deserves to be published in a present form.**

Many thanks for reviewing the manuscript and for your feedback.

**Reviewer #2: The paper “The critical factor: the role of quality in the performance of supported accommodation services for complex mental illness in England” tries to define critical quality factor for better efficiency evaluation and set up decision support system help in resource allocation.
My comments and concerns regarding the paper are given as follows.**

**Major comments:
1. The authors use DEA as a method of measuring RTE of supported accommodation services as part of health care system by employing commonly used input/outputs with addition of quality variables. However, the procedure and purpose of using all three methods is not clear and justified enough. Especially, it is not clear why and how authors use randomization of the original data (understanding the environmental and structural uncertainty and randomness). Is it objective to multiply number of DMU and why? How many DMUs are in the final dataset that has been used in DEA assessment?**

Thank you very much for your comments. We have answered your questions separately and propose the corresponding changes in the paper (in yellow).

- Relative Technical Efficiency (RTE) is a common and robust indicator for assessing the performance of any kind of comparable decision making units (in our case, mental health services). RTE is always concerned by the balance between the inputs consumed and the outputs produced, obviously looking for the best one. In order to clarify this concept we modified the introduction:

*The standard performance assessment of any kind of comparable service includes studying their input consumption and output production (directly related to the inputs consumed). These inputs/outputs are mainly technical, and in the end, the best service performance is always related to the most appropriate balance between the available inputs and the produced outputs. Researchers and decision-makers can seek to reduce (minimize) the amount of inputs for a given amount of outputs (input orientation) or vice versa to increase (maximize) the output production for a specific amount of inputs (output orientation).*

- In order to assess RTE we have used a non-parametric set of robust techniques that has been widely used in research and management: Data Envelopment Analysis (DEA) following the input orientation with variable return to scale as it was described in section “Decision Support System”. This technique has three main drawbacks: first, if the number of original observations (services) is not big enough DEA could not be discriminative enough (all the services would be efficient), second, it cannot understand uncertainty and randomness and, finally, it is completely blind and raw data values have to be interpreted according to a expert perspective in order to avoid wrong results. These drawbacks have to be solved when the EDeS-MH (Efficient Decision Support-Mental Health) decision support system was designed (see references number 19 and 21 for more detailed technical explanations).
- When the number of observations are less than the multiplication of the number of inputs by the number of outputs by 2 (2xNºinputsxNºoutputs), DEA can be no discriminative enough. Here the baseline scenarios have 3 inputs and 3 outputs so we need at least 18 services or more. When quality variables are included, we need, at least, 2x3x4=24 services. Surveyed residential services (9 move on services and 13 non-move on services) are not enough to carry out DEA in an standard way. On the other hand, the number of services 34 supported housing and 30 floating outreach ones can be considered likely scarce. Taking into account that all raw data should be considered under uncertainty (for example, if a specific service has 13 places it doesn’t mean that sometimes, due to more or less expected or unexpected reasons, it can have 12 or 14 or …) this is especially true when population, budged, etc. variables are included in the analysis (directly or for calculating rates).

Uncertainty plays a relevant decisional role and it was managed by the Monte-Carlo simulation engine (more technical explanations can be seen in references 18, 19, 21 and 32). In order to carry out this statistical technique, we have to transform raw data in statistical distributions (one for each data value). We have selected standard triangular ones with small variations at the left and right hands of the modal value that was the original data value (see section “Randomization …”). When the simulation process is run, the number of observations is multiplied (Nºsimulations by Nºobservations, for example: 500 simulations by 9 move on residential services = 4,500 observations to be analysed). Simulation makes an automatic sensitivity analysis by varying original values at random according to the selected statistical distribution. This process solves the two first drawbacks (the third one is explained in the following section in this document). In order to explain this process better we have included the following sentences in the paper:

*Nine move-on residential services, 13 non-move-on residential services, 34 supported housing services and 30 floating outreach services were finally analysed because they have complete quality datasets. Five hundred simulations were run by the DSS.*  *(“Basic statistics” section)*

*The simulation engine was developed to address the uncertainty (data imprecision and vagueness) and randomness (unexpected facts) of real environments and to artificially multiply the number of observations (29). The inner uncertainty of any ecosystem can be overcome by transforming original data values into statistical distributions (from standard datasets to statistical distribution bases). In each simulation, the Monte Carlo simulation engine analyses a new dataset selected at random. The statistical analysis of the final results (the process is stopped when the statistical error is lower than 2.5% for the mean) includes a sensitivity analysis of the* *ecosystem under study. The results (RTE scores) for each DMU and scenario are statistical distributions that can be studied in a more (basic statistics) or less (stability and entropy) standard manner (19,21). The characteristics of these statistical distributions represent the potential reaction of the DMU to data changes.(“Decision Support System” section)*

*… Second, quality variables must then be included as outputs to investigate the influence of quality on performance. Finally, when the number of observations is low (here, the number of move-on residential services is especially low), DEA cannot be sufficiently discriminative (in the end, the methodology tends to show that all DMUs are efficient). However, as explained before, the uncertainty analysis (Monte Carlo simulation engine) multiplies the number of observations by the number of selected simulations, which overcomes this DEA drawback. (“Decision Support System” section)*

**2. There is no theoretical fundaments of used methods (DEA, Fuzzy interference and Monte Carlo) given.**

Sorry and thank you very much for this comment that helps us to improve the paper. Again We have answered your questions separately and propose the corresponding changes in the paper.

- (Same explanation of the previous section). Relative Technical Efficiency (RTE) is a common and robust indicator for assessing the performance of any kind of comparable decision making units (in our case, mental health services). RTE is always concerned by the balance between the inputs consumed and the outputs produced, obviously looking for the best one. In order to clarify this concept we modified the introduction:

*The standard performance assessment of any kind of comparable service includes studying their input consumption and output production (directly related to the inputs consumed).* *These inputs/outputs are mainly technical, and in the end, the best service performance is always related to the most appropriate balance between the available inputs and the produced outputs. Researchers and decision-makers can seek to reduce (minimize) the amount of inputs for a given amount of outputs (input orientation) or vice versa to increase (maximize) the output production for a specific amount of inputs (output orientation).*

- Data Envelopment Analysis (DEA) is a set of well known non-parametric techniques to assess RTE (se references 19 and 21 for more technical details). In this paper we used input-oriented DEA with variable returns to scale. In order to clarify these aspects we have included the following sentences in the paper:

*… In this research, the* *variable returns to scale DEA (31) was selected because when studying MH services, real output variations cannot be considered proportional to the corresponding input modifications (32), and constant returns to scale would involve a constant variation that cannot be considered realistic. The input-oriented DEA model was applied to assess whether service input consumption can be reduced while assuming a constant output level (9), which* *is especially relevant for decision-makers who must allocate finite resources to meet population needs. Output maximization (output-oriented DEA) is especially difficult and sometimes not recommendable (for example, when the system artificially tries to maximize the number of users who are moved to a service with greater independence, this can be mathematically correct but from a health care perspective it has no sense at all) when supported accommodation services are assessed. (“Decision Support System” section).*

- Monte-Carlo simulation was used to include uncertainty into the analysis. This procedure allows us to develop a sensitivity analysis on the variables and scenarios. In the end, results are always statistical distributions where probabilities (to be efficient, to have an efficiency greater than 0.75, stability, entropy, etc.) are fundamental. In order to clarify this, we have included the following sentences into the paper.

*…under study. The results (RTE scores) for each DMU and scenario are statistical distributions that can be studied in a more (basic statistics) or less (stability and entropy) standard manner (19,21). The characteristics of these statistical distributions represent the potential reaction of the DMU to data changes*. *(“Decision Support System” section).*

- The fuzzy inference engine has been developed to interpret variable values according to a specific paradigm. In this paper the Balanced of Care model has been selected as this paradigm. All the variable values from the Monte-Carlo simulation engine are interpreted in terms of adequacy. For example, a low annual budget per place/bed is considered bad (not adequate) but too much budget per place/bed is also bad (not adequate), here the most appropriate values are located in between a range that is defined by the experts. Another example, a low quality score is bad and, in this case, the greater the value the greater the adequacy (greater values are always good). The fuzzy inference engine includes a knowledge-base formalized by standard IF … THEN rules. For example, IF the variable value of X is greater than a specific values THEN the value has to be transformed according to a mathematical equation or a fuzzy operator (product-sum gravity method). Our fuzzy inference engine transforms original data values (from the Monte-Carlo engine) into “interpreted” values according the information given by the experts (more technical details can be found in references 19 and 21). Trying to clarify this, we have included the following sentences into the paper.

*…when inside the range. The specific references for data value interpretation have been defined by a panel of experts according to the paradigm selected and their expertise. This process followed the EbCA model (5), where an iterative sequence of expert-based reviews culminates in a consensus. Once the references for interpreting variable values are defined, the EDeS-MH automatically runs a mathematical transformation based on an equation (linear monotone transformation) or a fuzzy operator (product-sum gravity method) to obtain the “transformed” value (21). These transformed values will be analysed by DEA to determine the corresponding RTE scores (statistical distributions). (“Decision Support System” section).*

**Minor comments:
3. The paper is not flawlessly written. It is a bit difficult to follow the line and purpose of paper.**

Thanks again for your comment. We have modify the aim of the paper in order to clarify the purpose of our research.

We include these sentences in the introduction:

- *… investment by providing relevant information for service managers to inform practice and service planning. Accordingly, this paper first presents a description of the ecosystem under study (a representative sample of supported accommodation services in England). Then, the selected variables are described and grouped into scenarios to highlight different perspectives of the ecosystem situation. Finally, the methodology used to assess ecosystem performance (including quality domains) is briefly described.*

The “Procedure” section has been completely rewritten in order to improve the readability of the paper.

**4. It is not necessary to have words Introduction, Objectives, Method, Results and Conclusion in the abstract. It is clear from the text what the purpose of those parts in abstract are.**

Many thanks, we have deleted these words in the abstract.

**5. In the Introduction, it says „Relative Technical Efficiency (RTE) is a decision support technique …”. This statement is not correct since Relative Technical Efficiency is a measure not technique.**

Many thanks, we have corrected it.

**6. “DEA can be included in a Monte Carlo simulation engine to include uncertainty and randomness in data values (all of them are considered statistical distributions) and design more realistic models (5).” This statement is not clear. Please explain how the DEA is included in a Monte Carlo Simulation engine (randomizing the data values or RTE).**

Thanks again for your comment. In almost any ecosystem raw data should be considered under uncertainty (for example, if a specific service has 13 places it doesn’t mean that sometimes, due to more or less expected or unexpected reasons, it can have 12 or 14 or …) this is especially true when population, budged, etc. variables are included in the analysis (directly or for calculating rates). Variable values were also obtained in a specific time and, if a wider perspective of the situation is required, researchers have to include some variability in order to be relatively sure (a probability) that the analysis can match (more or less) to a more actual situation. In order to clarify this, we have included the following into the paper:

*Original data were randomized using symmetric triangular statistical distributions (5% variation on each side of the corresponding original value). This range includes feasible data variations (imprecision and vagueness). No critical stress on the ecosystem was included in the analysis (randomness). This procedure includes both data variations corresponding to ecosystem evolution (population, user mobility, etc.) and the effect of the time. (“Procedure” section).*

**7. The meaning of the following sentence after Table 6 is not clear: “This is especially relevant in some services that showed lower RTE on average: 13, 18 and 21 (33.3%, Table 3)”; What is 33.3% referred on? There are similar examples in the whole section.**

You are completely right, sorry again. These percentages are calculated on the number of surveyed services in each type. The paragraphs have been modify accordingly.

*Generally, the inclusion of quality domains highlights a neutral-positive or positive impact on the performance of supported accommodation services in England, which is especially relevant in some services that showed a lower average RTE: 13, 18 and 21 (33.3% of the surveyed services, Table 3); 14, 90, 96 and 124 (30.8% of the surveyed services, Table 4); 18, 22, 27, 58, 70, 97, 110 and 115 (23.5% of the surveyed services, Table 5); and, finally, 1, 7, 36, 56, 87, 109, 121 and 122 (26.7% of the surveyed services, Table 6). In these services, the manager´s perception of quality provided by their services surpasses technical results (baseline scenario), probably because of non-evaluated variables or circumstances in care provision.*

*On the other hand, quality variables decrease service performance only in supported accommodation services 15, 31, 32, 86 and 98 (14.7% of the surveyed services, Table 5).*

*…* *Services where the inclusion of quality variables results in a neutral behaviour of their performance constituted the majority, respectively: 66.7%, 69.2%, 61.8% and 73.3% of the surveyed services. In these cases, the manager´s perception of quality provided by their corresponding services matches their technical results; they obtain a quality according to their resources and outcomes.*

**8. There is no straight-forward fuzzy inference interpretation and it importance explained in discussion or conclusion.**

Thank you very much. We have added the following sentences in the “Discussion” section.

*… accommodation services (34). The knowledge base is the core of the fuzzy inference engine because it provides expert-based information for transforming the original data values (from the Monte Carlo simulation engine) according to the balanced care model. This transformation is based on experts’ opinions on the adequacy of each data value and represents a specific framework that must change throughout the time span and depend on the socioeconomic environment. Therefore, the same dataset (raw data) can result in different results according to experts’ perceptions of reality. The fuzzy inference engine can “understand” the nuances that decision-makers assume in system management.*
