# Supplementary material for: The critical factor: The role of quality in the performance of supported accommodation services for complex mental illness in England
Source: PLoS One. 2022 Mar 17;17(3):e0265319. doi: 10.1371/journal.pone.0265319 (PMC8929565; doi:10.1371/journal.pone.0265319)
Supplement: Attachment — Submitted filename: Response to Reviewers.docx [file pone.0265319.s003.docx]

**Response to Reviewers**

Dear Editor and Reviewers, many thanks for assessing this research and proposed your valuable comments. We have carried out all the changes proposed, which are highlighted in yellow in the “Revised Manuscript with Track Changes”. In this document we have developed a rebuttal letter that responds to each point raised.

**Journal Requirements

Please review your reference list to ensure that it is complete and correct. If you have cited papers that have been retracted, please include the rationale for doing so in the manuscript text or remove these references and replace them with relevant current references. Any changes to the reference list should be mentioned in the rebuttal letter that accompanies your revised manuscript. If you need to cite a retracted article, indicate the article’s retracted status in the References list and also include a citation and full reference for the retraction notice.**

Many thanks for the comment. We have reviewed the citations and references, and we have deleted the link (<http://www.ucl.ac.uk/quest>) which was in “Methods>Setting” because it was not working. In addition, we have added a relevant reference for this sentence (in Methods>Setting) (highlighted in yellow in the manuscript with track changes):

“*Data for supported accommodation services from 14 nationally representative local authorities in England were collected for the QuEST study, which was funded by the National Institute of Health Research (2012-2017) (4)*.

**Reviewers’ comments**

**Reviewer #1: The authors have addressed the point of my concern. I am happy with their corrections. Hence, I would like to recommend this manuscript to be published.**

Dear reviewer 1, many thanks for your evaluating this research and providing your valuable comments.

**Reviewer #2: The authors addressed improved paper and addressed all the comment from the previous review round. I would like to recommend to authors to include to DEA mathematical models used in the paper.**

Dear referee, the DEA model has been included in section “Methods>Decision Support System” as follows (highlighted in yellow in the manuscript with track changes):

“*The standard DEA model is a linear programming one which structure is:*

$$\begin{matrix} Min\theta-\varepsilon\left(\sum_{h=1}^{i} S_{h}^{-}+\sum_{r=1}^{j} S_{r}^{+}\right) \\ s.t. \\ \sum_{m=1}^{d} x_{hm}\lambda_{m}+S_{h}^{-}=\theta x_{ho};h=1,2,...,i \\ \sum_{m=1}^{d} y_{rm}\lambda_{m}-S_{r}^{+}=y_{ro};r=1,2,...,j \\ \sum_{m=1}^{d} \lambda_{m}=1 \\ \lambda_{m}\geq0;m=1,2,..,d \end{matrix}$$

*where d is the number of DMU, i the number of inputs and j the number of outputs. The DMU m consumes x_hm_ of input h and produces y_rm_ of output r. θ is the efficiency score and* $S_{h}^{-}$ *and* $S_{r}^{+}$ *are the slacks (21)”.*
